# Use of Human Intestinal Enteroids to Evaluate Persistence of Infectious Human Norovirus in Seawater

**DOI:** 10.3201/eid2807.220219

**Published:** 2022-07

**Authors:** Marion Desdouits, David Polo, Cecile Le Mennec, Sofia Strubbia, Xi-Lei Zeng, Khalil Ettayebi, Robert L. Atmar, Mary K. Estes, Françoise S. Le Guyader

**Affiliations:** Institut Franҫais de Recherche pour l’Exploitation de la Mer, Nantes, France (M. Desdouits, D. Polo, C. Le Mennec, S. Strubbia, F.S. Le Guyader);; Baylor College of Medicine, Houston, Texas, USA (X.-L. Zeng, K. Ettayebi, R.L. Atmar, M.K. Estes)

**Keywords:** Norovirus, persistence, stability, human intestinal enteroids, marine environment, viruses, enteric infections, food safety, France, United States

## Abstract

Little data on the persistence of human norovirus infectivity are available to predict its transmissibility. Using human intestinal enteroids, we demonstrate that 2 human norovirus strains can remain infectious for several weeks in seawater. Such experiments can improve understanding of factors associated with norovirus survival in coastal waters and shellfish.

Human noroviruses are the major cause of viral gastroenteritis worldwide (*1*) and the most common cause of foodborne or waterborne outbreaks in Europe (*2*). Noroviruses spread through fecal–oral transmission, mainly person to person, but also spread through environmental contamination (*1*). Food and drinks can be contaminated by infected food handlers and, during production, by human sewage spillover (*3*). When grown in contaminated seawater, filter-feeding shellfish bioaccumulate human noroviruses in their tissues (*2*,*4*). Shellfish, especially those eaten raw, are among the main foods involved in foodborne epidemics (*2*,*5*).

Noroviruses are diverse, positive-stranded RNA viruses, classified into >10 genogroups (G) and many genotypes; most noroviruses that infect humans belong to genogroups GI and GII (*6*). Since 1995, the epidemiology of human noroviruses has been dominated by the GII.4 genotype (*1*). Of note, GII.4 appears to be predominantly transmitted person to person, whereas other genotypes, such as GII.6, GII.3, and some from GI, are more often implicated in foodborne or waterborne outbreaks (*1*–*5*). This difference might reflect variations in particle resistance to environmental conditions (*1*,*7*), but empirical data are lacking.

The small, nonenveloped human norovirus particles are considered very stable outside their host, especially in aquatic environments (*1*,*7*,*8*). Particles are also highly infectious, leading to human infection even when very low amounts of virus are present in shellfish (*9*). Yet, for almost 50 years, the lack of a reproducible cell culture system impaired the direct assessment of human norovirus infectivity in environmental conditions. Hence, data used for risk assessment rely on molecular assays or surrogate viruses (*2*). Previously, we used a surrogate calicivirus, Tulane virus (TuV), to estimate the persistence of infectious human norovirus in shellfish (*10*). However, because surrogates might underestimate the actual stability of human norovirus (*11*) and do not enable comparisons between different norovirus strains, direct assessments of infectivity in the environment and foods are needed to learn more about foodborne transmission and design optimal sanitary regulations (*2*).

Since 2016, human intestinal enteroids (HIEs) have enabled the in vitro cultivation of many human norovirus strains and represent a physiologically relevant model to assess whether the virus is infectious (*12*–*15*). In this study, we used HIEs to evaluate the persistence of infectious human norovirus in natural seawater, the last matrix before bioaccumulation by shellfish, in comparison with TuV.

## The Study

**Figure 1:**
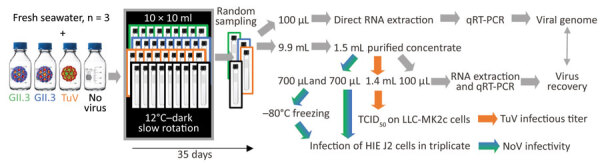
Study design on use of HIEs to evaluate persistence of infectious human norovirus in seawater. Comparison of the stability of 2 human norovirus strains (GII.3 indicated by green, GII.4 indicated by blue) and TuV (orange) in seawater. We conducted 3 independent experiments with different fresh seawater samples. Spiked seawater (120 mL) was split in 10 mL aliquots in glass tubes, incubated at 12°C in the dark under constant rotation (10 rpm), and randomly sampled once or twice per week for 5 weeks (35 days). Grey arrows indicate steps or treatments applied to all samples; blue-green arrows indicate steps or treatments applied to human norovirus and control without virus; orange arrows indicate steps or treatments applied to TuV only. HIE, human intestinal enteroid, NoV, norovirus; qRT-PCR, one-step quantitative reverse transcription PCR; TCID_50_, 50% median tissue culture infectious dose; TuV, Tulane virus.

We compared the stability in seawater of 2 human norovirus strains, GII.4 (TCH11-64) and GII.3 (TCH04-577), obtained from human stool filtrates as described previously (*12*), and 1 TuV strain (M33) produced in simian LLC-MK2 cells (*10*). Ethics approval for collection of virus-containing fecal samples and human intestinal cells was obtained from the Baylor College of Medicine Institutional Review Board. We conducted 3 experiments with fresh samples of natural seawater (Table 1). We used viral stocks to spike 120 mL of seawater, which we then split into 10 mL-aliquots and incubated at 12°C in a thermostatic cabinet (Memmert, https://www.memmert.com) (Figure 1). Once or twice a week, we randomly sampled an aliquot, extracted nucleic acids from 100 µL by using the NucliSens kit on a MiniMag (bioMérieux, https://www.biomerieux.com), and assessed the viral genome concentration by quantitative reverse transcription PCR (*10*).

**Table 1:** Characteristics of seawater samples used for 3 experiments using human intestinal enteroids to evaluate persistence of infectious human norovirus

Experiment	1	2	3
Collection date*	2018 Sep 5	2018 Oct 16	2019 Apr 30
Physio-chemistry			
Salinity, %	36.5 (35†)	35	33.3
Turbidity, NTU	0.67	7.50	1.14
pH	7.8	7.9	7.9
Total suspended solids, mg/L	4.0	3.0	1.0
Dissolved organic carbon, mg/L	2.3	1.6	2.1
Phosphate, mg/L	0.079	0.082	0.192
Nitrate, mg/L	0.4	0.3	0.8
Microbiology			
Total nonmarine bacteria/100 mL	100	>300	37
Total marine bacteria/100 mL	>300	>300	>300
* Escherichia coli*/100 mL	0	0	0

*Coastal seawater samples were collected and sand-filtered at the same experimental shellfish farm at different dates, kept at 4°C, and used within 1 week of collection. NTU, Nephelometric Turbidity Units. †Salinity of seawater was adjusted to 35% using distilled water for experiment 1.

**Figure 2:**
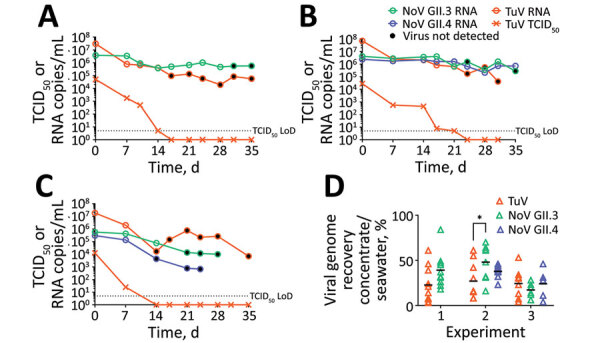
Persistence of viral RNA and infectious norovirus and Tulane virus in seawater. Concentration of viral RNA measured by quantitative reverse transcription PCR (qRT-PCR) in seawater (circles, RNA copies/mL), and of infectious TuV measured by TCID_50_ (cross, TCID_50_/mL), during experiments 1 (A), 2 (B), and 3 (C). Open circles mark the detection of infectious virus on HIE cells (human norovirus) or through TCID_50_ on LLC-MK2 cells (TuV). Black circles indicate the absence of infectious virus detection. Dotted lines indicate the theoretical LoD of the TCID_50_ assay (5 TCID_50_/mL). D) Recovery of the viral genome after purification and concentration steps, defined as the ratio (%) of viral genome in the concentrate to that in the seawater, as measured by qRT-PCR, for each virus and time point during the 3 experiments. Black lines indicate the mean per experiment and virus. Recovery was not statistically different between experiments and viruses except for TuV and norovirus GII.3 during experiment 2 (analysis of variance, Sidak’s multiple comparisons test; *p = 0.0318) (GraphPad Prism version 9.2.0, https://www.graphpad.com/scientific-software/prism). LoD, limit of detection; NoV, norovirus; TCID_50_, 50% median tissue culture infectious dose; TuV, Tulane virus.

During experiments 1 and 2, the genomic concentration of human norovirus GII.3 remained highly stable; 0.8 log_10_ losses in experiment 1 and 1.2 log_10_ losses in experiment 2 occurred over 5 weeks (Figure 2, panels A, B). We did not assess GII.4 virus in experiment 1, but we observed similar stability at the genomic level in experiment 2, a 0.5 log_10_ decrease (Figure 2, panel B). During experiment 3, GII.3 and GII.4 genomic concentrations were >1 log_10_ lower than the other experiments at day 0 and reached a total loss of 1.8 and 2.7 log_10_ over 4 weeks (Figure 2, panel C). For the 3 experiments, TuV genomic levels were higher than human norovirus at day 0 but decreased more quickly; total losses of 2.7 (experiment 1), 3.2 (experiment 2) and 3.4 (experiment 3) log_10_ occurred over 5 weeks (Figure 2, panels A–C), consistent with the decay rate observed previously in contaminated oysters (*10*).

For the remainder (9.9 mL) of the seawater aliquots, we filter-sterilized, concentrated by centrifugal ultrafiltration, and desalted, adapting a method used to purify infectious TuV from oysters (*10*). To verify efficacy, we used 100 µL of purified concentrate for RNA extraction to quantify the viral genome and to calculate the proportion of virus recovered in the concentrate compared with the proportion of virus in seawater (Figure 1). For all experiments combined, viral recovery ranged from 4% to 61% for TuV, 6% to 70% for GII.3, and 4% to 37% for GII.4 (Figure 2, panel D). The recovery of human norovirus tended to be even higher than for TuV, especially in the case of GII.3 in experiment 2 (Figure 2, panel D).

We used purified concentrates of TuV to assess its infectious titer through 50% tissue culture infectious dose on LLC-MK2 cells (*10*) (Figure 1). Infectious TuV was detected for 14 days during experiment 1 and for 21 days during experiment 2 (Figure 2, panels A, B), similar to the length of detection in contaminated oysters (*10*). Experiment 3 also showed a faster loss of infectious TuV, which was not detected after 7 days (Figure 2, panel C).

We used the purified concentrates of human norovirus to infect differentiated jejunal J2 HIE monolayers in triplicate (*12*), either upon collection or after storage at −80°C (Figure 1). The geometric mean fold increase (GMFI) in viral genome was measured between 1 hour and 72 hours after infection; the virus was considered infectious when GMFI >3.0 (Table 2). We detected infectious human norovirus GII.3 at up to 28 days (experiment 1), 31 days (experiment 2), and 14 days (experiment 3); infectious norovirus GII.4 was recovered throughout the 35 days in experiment 2 and through day 7 in experiment 3 (Table 2). Progressive loss in human norovirus infectivity is suggested by the GMFI decrease during all experiments for both viruses (Table 2). Of note, for all experiments, infectious GII.3 and GII.4 were detected for longer periods of time than infectious TuV (Figure 2, panels A–C), suggesting that human norovirus is more stable than TuV in seawater, especially because the initial concentrations of TuV were higher (Figure 2, panels A–C). Our results also suggest that the persistence of GII.3 and GII.4 is similar in these settings, but this finding needs further validation with a quantitative assay, because J2 HIE monolayers are more susceptible to GII.4 than GII.3 (*12*). Indeed, we observed the absence of infectious human norovirus when input genome levels were close to the sensitivity threshold of the assay (2 × 10^4^ for GII.3, 1.2 × 10^3^ for GII.4) (*12*), which suggests that infectious human norovirus particles might still have been present but were undetected. Finally, all virus data show that experiment 3 differs from the 2 others, which could have been caused by uncharacterized variables of the different seawater samples.

**Table 2:** Detection of infectious human norovirus GII.3 or GII.4 in 3 experiments using HIEs to assess persistence of infectivity*

Time	Experiment 1			Experiment 2						Experiment 3				
	GII.3			GII.3			GII.4			GII.3			GII.4	
	Inf	GMFI†		Inf	GMFI†		Inf	GMFI†		Inf	GMFI†		Inf	GMFI†
0	+	267, 1,082,‡ 103§		+	496		+	1,290		+	639, 600§		+	429, 845§
7	+	248, 787‡		+	293		+	476		+	486, 205§		+	3.2, 50§
10	+	31, 2.0‡		ND			ND			ND			ND	
14	+	366, 10,‡ 8.2§		+	31§		+	151§		+	3.2, 0.4§		–	No Ct
17	+	6.7, 1,‡ 12§		+	53§		+	139§		–	No Ct		–	No Ct
21	+	12, 13,‡ 4.2§		+	70§		+	102§		–	No Ct		–	No Ct
24	+	3.1, 15.7,‡ 3.1§		+	3.1§		+	46§		–	No Ct		–	No Ct
28	+	0.5, 83,‡ no Ct§		+	213		+	3.7		–	No Ct		–	No Ct
31	–	0.7, no Ct‡		+	6.1§		+	16§		–	No Ct		–	No Ct
35	–	0.9, 1.0‡		–	0.8§		+	25§		–	No Ct		–	No Ct

*No ct indicates GMFI could not be calculated because norovirus genome could not be detected by quantitative reverse transcription PCR at 1h or 72h postinfection or both. Ct, cycle threshold; G, genotype; GMFI, geometric mean fold increase; HIE, human intestinal enteroids; Inf, infectious; ND, not done: +, positive (detected); –, negative (not detected). †Each value represents the GMFI in human norovirus genome copies, between 1h and 72h post-infection, in triplicate wells of HIE cultures (n = 3). Evidence of replication was defined as a GMFI >3.0. Freshly prepared and undiluted viral concentrates were used to infect HIE in most experiments and time points, expect for experiment 2 where HIE cultures died after day 7. **‡**Fresh viral concentrates diluted 1/10 in culture medium were also used in experiment 1. **¶**To assess the possibility to freeze viral concentrates before the HIE infectivity assay, pure viral concentrate stored frozen at −80°C for several weeks and thawed once for culture on HIE were also used in some assays and showed results similar to those with fresh concentrates in 10 out of 11 tests.

## Conclusions

This study demonstrates that HIEs can be used to study infectious human norovirus persistence in seawater, an environmental matrix, and confirms the virus’s high stability. Using 3 natural seawater samples, we observed persistent yet variable viability of human norovirus, showing that the nature of the seawater affects viral infectivity. This model will enable further research assessing possible factors at play, such as the bacterial flora or the physio-chemical parameters of the water. Together with data on foodborne outbreaks, this model will help determine the behavior of human norovirus in the environment and thus protect human health by enabling sanitary regulations to be adapted for actual infectious risks.
